# Supplementary figures and images for: The microbiome and metatranscriptome of a panel from the Sarracenia mapping population reveal complex assembly and function involving host influence
Source: Front Plant Sci. 2024 Oct 15;15:1445713. doi: 10.3389/fpls.2024.1445713 (PMC11518717; doi:10.3389/fpls.2024.1445713)

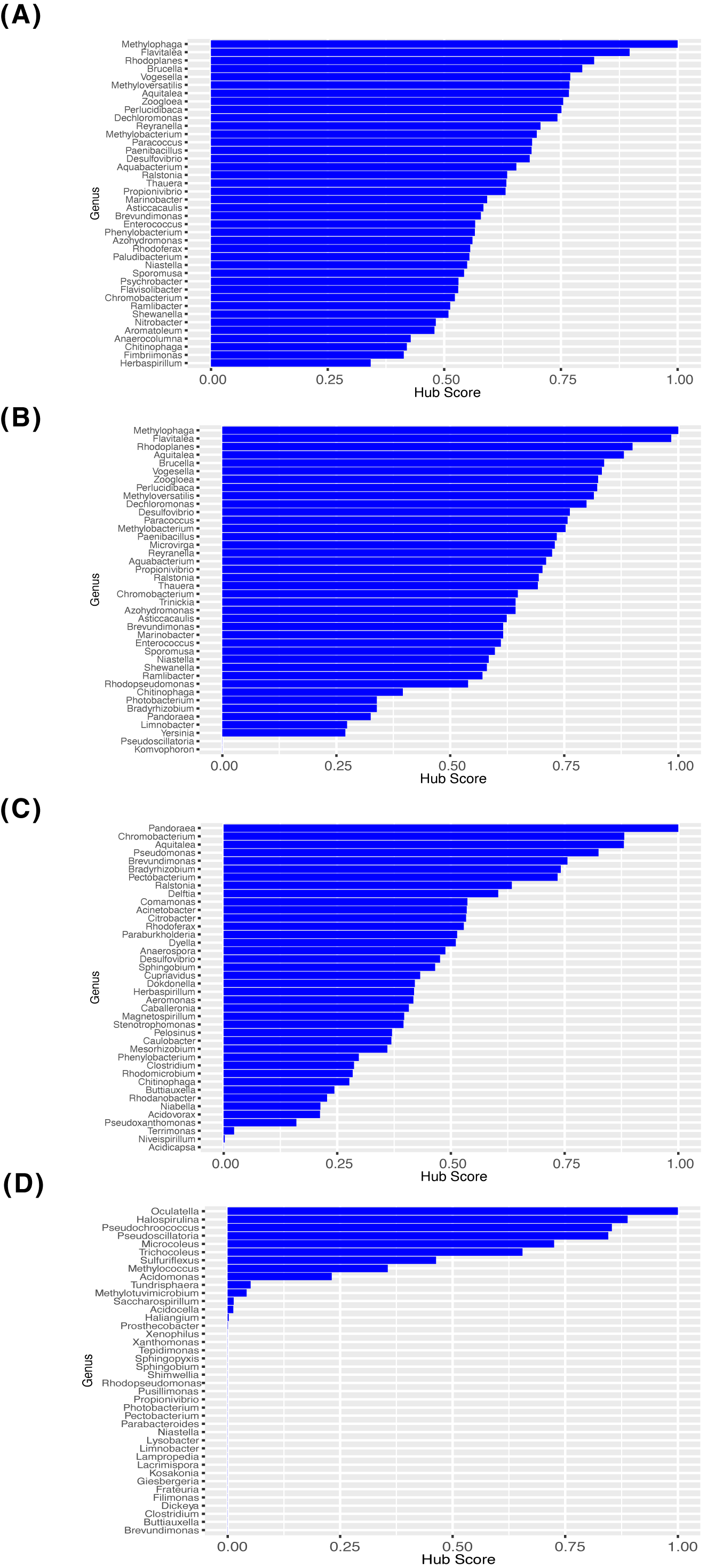

Supplement: Supplementary Figure 1 — Examining the changes in the network structure under various correlation, p-value, and node counts criteria. [file Image1.tif]
